# Cardiovascular disease risk reduction in rural China: a clustered randomized controlled trial in Zhejiang

**DOI:** 10.1186/1745-6215-14-354

**Published:** 2013-10-25

**Authors:** Xiaolin Wei, Guanyang Zou, Weiwei Gong, Jia Yin, Yunxian Yu, John Walley, Zhitong Zhang, Rebecca King, Kun Chen, Marc Ka Chun Chong, Benny Chung Ying Zee, Su Liu, Jinling Tang, Sian Griffiths, Min Yu

**Affiliations:** 1 School of Public Health and Primary Care, Chinese University of Hong Kong, Hong Kong, China; 2 Nuffield Centre for International Health and Development, University of Leeds, Leeds, UK; 3 Zhejiang Centre for Disease Control and Prevention, Hangzhou, Zhejiang, China; 4 School of Public Health, Zhejiang University, Hangzhou, Zhejiang, China

**Keywords:** Cardiovascular disease, Risk, Events, Randomized controlled trial, Primary care

## Abstract

**Background:**

Cardiovascular disease (CVD) is a major cause of death in China. Despite government efforts, the majority of hypertensive and diabetic patients in China do not receive proper treatment. Reducing CVD events requires long-term care that is proactive, patient-centred, community-based, and sustainable. We have designed a package of interventions for patients at high risk of CVD to be implemented by family doctors based in township hospitals (providers of primary care) in rural Zhejiang, China. This trial aims to determine whether the systematic CVD risk reduction package results in reduced CVD events among patients at risk of CVD compared with usual care, and whether the package is cost-effective and suitable for routine implementation and scale-up.

**Methods/Design:**

This is a prospective, open-label, cluster randomized controlled trial (RCT) with blinded data analysis. The trial will randomize 67 township hospitals with 31,708 participants in three counties in Zhejiang Province. Participants will be identified from existing health records and will comprise adults aged 50 to 74 years, with a calculated 10-year CVD risk of 20% or higher, or diabetes. In the intervention arm, participants will receive a package of interventions including: 1) healthy lifestyle counseling (smoking cessation, and salt, oil, and alcohol reduction); 2) prescription of a combination of drugs (antihypertensives, aspirin, and statin); and 3) adherence support for drug compliance and healthy lifestyle change. In the control arm, participants will receive usual care for hypertension and diabetes management at individual clinicians’ discretion. The primary outcome is the incidence of severe CVD events over 24 months of follow-up. All CVD events will be defined according to the World Health Organization (WHO) monitoring of trends and determinants in cardiovascular disease (MONICA) definitions, diagnosed at the county hospital or higher level, and reported by the Zhejiang surveillance system. Secondary outcomes include: mean systolic and diastolic blood pressure, blood glucose, serum total cholesterol (TC), and adherence to appointments, and drugs and lifestyle changes.

**Discussion:**

This trial focuses on risk reduction of CVD rather than specific diseases. It is not designed to compare therapeutic and healthy lifestyle interventions, but rather their combined effects in primary care settings. Through the trial, we intend to understand the effectiveness of the comprehensive CVD reduction package in routine practice. We also intend to understand the barriers and facilitators to implementing the package, and thus to advise on policy and practice change.

**Trial registration:**

Current Controlled Trials: ISRCTN58988083

## Background

Cardiovascular disease (CVD) accounts for 38% of total mortality in China [1]. The mortality rate for stroke in China is four to six times higher than that of Japan and the USA [2]. In 2008, China had 200 million people with hypertension, 160 million with dyslipidemia, 92 million with diabetes, and 200 million who were overweight [1,3]. CVD events are predicted to increase by 23% from 2010 to 2030, resulting in an additional 21.3 million CVD events and 7.7 million deaths [4].

The majority of people in China with hypertension or diabetes are not aware of their disease(s) and do not get proper treatment: a national survey in 2002 showed that hypertension prevalence increased by 73% between 1991 and 2002; only 23% of hypertensive patients were aware of their status; of these only 17% were on treatment; and of those who were treated only 4% had their blood pressure under control [5]. A national survey in 2008 showed diabetes prevalence was 9.7%; however, the prevalence of diagnosed diabetes was only 2.7% [3]. A recent survey found hypertension prevalence in one rural area of China was 40% [6]. In China, patients with hypertension, diabetes, and related CVD conditions mostly receive *ad hoc* care, they are prescribed medications based on the family doctor’s knowledge, and many patients stop taking drugs once they feel well. Patient adherence to antihypertensive drugs is low, ranging from 20% to 60% [5,6]. The pilot study in Zhejiang suggested that 6% of rural residents aged between 40 and 75 years had a 10-year CVD risk of 20% or higher [7], and nearly half of hypertensive or diabetic patients had stopped their treatment because of a reduction in symptoms in the past year [7]. Service improvements and sustainable systems to encourage patient adherence to treatment and healthy lifestyles are therefore needed.

Meta-analysis has indicated that CVD risk reduction by treating hypertension or diabetes is not sufficient [8]. A comprehensive approach is needed, based on drug therapies and lifestyle support in primary care settings for patients with high CVD risk. Systematic reviews have shown that antihypertensive drugs will reduce the risk of stroke by 63% and ischemic disease by 46% [9], while statins reduce the risk of stroke and ischemic disease by 60% and 17%, respectively [10]. Meta-analysis confirms the safety of using aspirin, statins, and antihypertensive drugs, and that they can reduce CVD risk by 80% [11,12]. Two recent trials of a fixed-dose ‘polypill’ successfully reduced systolic blood pressure by 6 to 7 mmHg: the authors predicted that the polypill could reduce coronary heart disease by 62% and stroke by 48% in the long-term [13,14]. In addition to drugs, healthy lifestyle counseling has shown a small-to-moderate effect in reducing multiple CVD risk factors [15,16], improving patient quality of life [17], and reducing mortality [18]. A study in Ontario, Canada, reported that a community-based education program for older people reduced CVD-related hospital admissions in general by 9% within 10 weeks [19]. A review in China suggested community-based health education and management may reduce the stroke rate by 20% to 30% among the general older population over 3 to 4 years [20]. Salt reduction or substitution leads to reduced blood pressure [21], and a study in China reported that salt substitution for 12 months reduced systolic blood pressure by 3.7 mmHg [22]. Smoking cessation and increased exercise can be sustained for 10 to 20 years after the intervention [23,24].

Currently, large-scale community-based interventions, such as The Oxford Health Alliance’s Community Interventions for Health [25], are being implemented in several countries including China, but not as trials. A trial using village doctors to provide individual drug management and salt consumption reduction is on-going [26]. However, village doctors currently play a very limited role in chronic disease management due to their low capacity and because they practice as *de facto* private practitioners [27,28]. Furthermore, previous trials have mainly used blood pressure as the primary endpoint.

Based on information from the China hypertension control guideline and systematic reviews, we have developed a package of CVD risk reduction interventions including: 1) health education on smoking cessation, and salt, oil, and alcohol reduction; 2) drug therapy using antihypertensives, low dose aspirin, and a statin; and 3) adherence support to encourage drug compliance and healthy lifestyle change. The intervention package is designed to fit within China’s national package of essential public healthcare. This trial aims to determine whether the systematic CVD risk reduction package results in reduced CVD events among patients at risk of CVD compared with usual care, and whether the package is cost-effective and suitable for routine implementation and scale-up. We believe this trial is the first to use CVD event rates as the primary outcome; thus it will provide additional evidence to inform changes in policy and practice.

## Methods

### Design of the study

**Figure 1:**
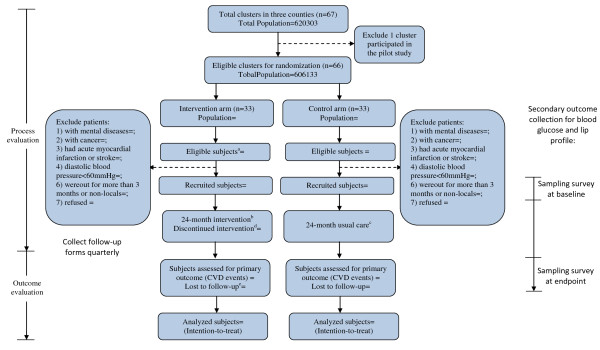
Trial design flow chart.

This will be a health service delivery trial that combines screening, treatment, and lifestyle change interventions. It is designed as a prospective, open-label, cluster randomized controlled trial (RCT) with blinded data analysis (Figure 1). We conducted two pilot studies prior to the trial: a pilot study to assess the feasibility of implementing the CVD prevention guidelines in two township hospitals in Zhejiang; and a pilot implementation in one township hospital from September 2012 to June 2013 to examine the feasibility and acceptability of intervention and research procedures. The results of the two pilot studies have been used to inform the development of the forthcoming trial.

### Setting

The trial will take place in three counties of Shaoxing Prefecture in Zhejiang Province. Zhejiang is located on the east coast of China and has a population of 54 million. CVD is the largest cause of death in Zhejiang: the population-based percentage of deaths due to CVD was 1.8% in Zhejiang [29] in 2009, as compared to 2.0% in China in 2007 [1]. The prevalence rates of the major CVD risk factors is similar between Zhejiang and China: for example, the hypertension prevalence was 20% in Zhejiang [29] and 19% in China [1]; and the prevalence of diagnosed diabetes was 4.1% in Zhejiang in 2009 [29], similar to that in China.

Primary care is provided by public township hospitals. Each township hospital covers 50,000 to 200,000 people. Family doctors in the township hospitals are responsible for acute and preventive care, including chronic disease control, with an average population of 1,500 per family doctor. Since 2009, Zhejiang has established a chronic diseases surveillance system, covering all the counties in the province. Each health facility must report any CVD events to the Centre for Disease Control and Prevention (CDC) through this system. This system is checked daily in CDCs at the provincial, prefectural, and county levels, allowing easy collection of CVD events within the administrative boundaries, and elimination of any duplication of CVD events reported by different health facilities.

### Inclusion and exclusion criteria for participants

Inclusion criteria for clusters are: township hospitals that have electronic health records of their residents for the last two years; are situated within the three selected counties of Zhejiang; and have agreed to participate in the trial.

CVD risk will be assessed at enrolment by the Asian Equation. The Asian Equation was developed using six cohorts of 172,077 individuals who were followed-up for 20 years in several Asian countries [30], and evaluated using independent Chinese cohorts. Its discrimination ability is better than the Framingham Equation. The Asian Equation calculates an individual’s risk of having any CVD event in the next 8 years based on gender, age, systolic blood pressure, total cholesterol (TC), and smoking status, shown as a percentage from 0% to 100%. In this study, we revised the Asian Equation to estimate the CVD risk in the next 10 years to be comparable with other internationally recognized equations. The Asian Equation effectively predicts both ischemic and hemorrhagic CVD events in the Chinese population, while others only predict ischemic CVD events [31,32]. TC is part of the health check of all residents aged over 60 years in China. Patients with diabetes are automatically considered at high risk of CVD [33]. Existing health records will be used to identify participants with a high CVD risk using the Asian Equation and participants with diagnosed diabetes.

Inclusion criteria for participants are: adults aged 50 to 74 years who have provided informed consent; hold permanent residence in the study area; and have a calculated 10-year CVD risk of 20% or higher, or a recorded medical history of diabetes. Diabetic patients are included because diabetes is a single decisive factor for high CVD risk [33].

Exclusion criteria for trial participants are: patients with mental health problems or other disabilities which mean they cannot communicate with family doctors well or regularly; patients who have had any severe CVD event, including acute coronary events, acute myocardial infarction, and any ischemic or hemorrhagic cerebrovascular events (identified from the chronic diseases surveillance system), as they are managed by county hospitals, and often do not seek care in township hospitals. We will also exclude: patients with other severe diseases, for example, late stage cancer; patients who are hospitalized; patients who have had serious adverse effects to the drugs used in the trial; patients who have diastolic blood pressure lower than 60 mmHg as they are contraindicated to hypertensive drugs; patients who are at high risk of CVD but do not have hypertension (more than 140/90 mmHg) or diabetes, because they are not routinely treated in township hospitals (although they will be referred for healthy lifestyle consultation, and further diagnosis if appropriate); and individuals who decline to participate in the trial.

All study participants will be treated and managed under their township hospital by their family doctor, who is responsible for the chronic disease management of one village. The family doctors will ensure that all eligible participants from their catchment areas are seen and recruited at baseline and followed-up.

### Usual care

In the control arm, usual hypertension and diabetes management will continue according to current national guidelines: conventional clinical consultations; treatment provision according to existing knowledge and at the individual clinician’s discretion; no specific adherence support; and non-systematic health education. Within usual care, treatment and lifestyle changes are recommended to participants based on either hypertension or diabetes, but not based on the holistic approach of CVD risk reduction. Patients with both hypertension and diabetes often need to attend different consultations for medications and lifestyle advice.

### Intervention

In the intervention arm, participants with a CVD risk of ≥20% and those with diagnosed diabetes will be provided, as appropriate, with a systematic CVD risk reduction package. Patient addresses and contact numbers are routinely recorded as part of usual care, and will be used to trace patients for follow-ups. The intervention activities are designed to fit within the job descriptions of family doctors and are detailed in a desk guide. The systematic CVD risk reduction package comprises: healthy lifestyle education, drug therapies, and adherence support.

#### Healthy lifestyle education

Regular health education consultations will be provided by township hospital family doctors and reinforced monthly during follow-up appointments. Consultations will include smoking cessation advice and support, as well as advice on healthy eating (especially salt and oil reduction) and advice on alcohol reduction. There is no charge for healthy lifestyle consultations.

#### Drug therapies

Drugs prescribed in the intervention will be from the essential drug list. All patients with hypertension will be prescribed with a combination of two antihypertensive drugs, a statin, and a low dose of aspirin. Patients only with diabetes will be prescribed one antihypertensive drug, a statin, and a low dose of aspirin, plus their anti-diabetic drugs. The antihypertensive drugs and aspirin cost around £0.50 per month. Statins are relatively expensive drugs, for example, simvastatin costs approximately £10 per month. In Zhejiang, the rural health insurance scheme covers 30% of outpatient costs. However, patients must pay the remaining 70% at each consultation. There is no user fee for patient consultations.

The desk guide to be used in the trial includes a clinical algorithm and information on the use of the recommended drugs, as well as how to replace existing drugs, if necessary. The guide includes a list of contraindications and adverse effects for aspirin, statins, and antihypertensives [12,34,35]. The safety profiles of the individual drugs recommended in the intervention are well established [11,36,37]. The trial will pose minimal risks to patients, since drugs will be modified on an individual basis.

#### Adherence support

Participants will meet with their family doctors monthly either in the township hospitals, health stations (branches of township hospitals), village clinics, or at a family doctor home visit. Participants will be referred to a nurse educator to improve drug adherence initially monthly and then quarterly. Patient support will draw on our experience using similar systems for tuberculosis and antiretroviral treatment adherence [38,39]. A treatment supporter will be chosen and, together with the patient, will be advised about their role supporting the patient and encouraging drug and lifestyle advice adherence. Expert patients will be trained as peer educators. Late attendees of clinical appointments will be reminded by mobile phone.

Primary care providers in the intervention arm will be trained on counseling skills, using role-play in their training. Refresher training sessions will be provided and feedback from interventions will be discussed in the monthly internal meetings of township hospitals. A quality control plan has been developed and embedded within the routine essential public health service.

### Outcomes

#### Primary outcome

The primary outcome is the incidence of severe CVD events over 24 months of follow-up. CVD events are defined according to the WHO MONICA definitions, including: 1) acute coronary events; 2) acute myocardial infarction, sudden cardiac death, and other deaths due to coronary and vascular disease; and 3) ischemic or hemorrhagic cerebrovascular events. Minor CVD events, such as chronic cerebral arteriosclerosis and transient ischemic attacks, are not included because these events do not usually end in hospitalization and are often not reported. Participants who cannot be contacted after three attempts (either by telephone, email, or letter) by 24 months after randomization will be deemed as lost to follow-up. Person years will be used to calculate CVD event rates for participants whose CVD event outcomes are recorded. We will also record the time of patient contacts in both arms. Since sufficient information about CVD events and contact times is available, the outcome of CVD events and contact times will cover the last contact time for the loss to follow-up cases and mortality cases that were not due to CVD events.

#### Sample size

The intervention is expected to lead to a reduction of at least 20% in CVD event rates within two years. This is based on a conservative estimate from the results from meta-analyses [9,11,18,37], a review of community-based programs in China [20], and recent trials [13,14,19,40]. According to the baseline of the pilot study, the target population (aged 50 to 74 years with ≥20% CVD risk and diabetes) has a CVD incidence rate of 5% within two years: we use this as an estimate of the CVD event rate in the usual care arm. In order to detect a 20% difference between the two arms with 90% power, using two-sided testing at the 5% level, and a moderate coefficient of variation (CV) of 0.15 [41] with approximately 450 participants per cluster (using harmonic means to adjust for imbalances in cluster size [42]), we estimate 32 clusters per arm are needed [41,43].

#### Secondary outcomes

The secondary outcomes are: 1) mean systolic and diastolic blood pressures of participants; 2) time to the first reported CVD event, mortality, and morbidity of CVD events during the trial period of 24 months; 3) mean change in blood glucose and glycated hemoglobin (Hb1Ac); 4) mean change in serum TC and low density lipoprotein; 5) adherence to booked appointments, using the denominator of all participants registered, including defaulters; 6) self-reported adherence to drugs and healthy lifestyle change, for example, smoking cessation rates (for participants who smoke at randomization, the percentage who smoke less than one cigarette a week at 24 months after randomization); 7) cost-effectiveness; and 8) feasibility measures.

### Sample size calculation for secondary outcomes of blood profiles

Due to resource limitations, we will collect participant blood samples to measure blood glucose, Hb1Ac, and lipid profiles only on a subsample of participants. The survey will be conducted at 0 and 24 months after randomization. The sample size is based on change in TC levels and the blood profile indicator is anticipated to show the smallest change of means (and hence expected to require the largest sample size). According to the pilot study, the mean and standard deviation of TC were 4.48 mmol/L and 0.8526 mmol/L, respectively. We anticipate a reduction of 6%. In other similar studies, a TC reduction of 16% to 20% was observed [14,44]. In the pilot study, a third of participants took statins. We therefore assume that we will achieve a third of 18% reduction in TC, that is, 6% in the intervention arm. We estimate that we need to increase the sample size by a factor of two to allow for clustering, with an intracluster correlation (ICC) of 0.01, and a maximal of 100 participants per cluster. Clusters will be chosen randomly from each arm and sampling will be undertaken within the required clusters. With 90% power and using two-sided testing at the 5% level, six clusters are required in each arm, allowing for a loss to follow-up rate of 10% [42,45].

### Randomization

All township hospitals with available health records in the three countries in Shaoxing, Zhejiang, will be selected except the pilot site. Written informed consent will be sought from each township hospital and from each of the participants in the township hospitals before screening.

Townships will be randomized, without stratification, to intervention or control in a 1:1 ratio. No blinding will be performed in this study. The sensitivity analysis shows that stratified randomization does not significantly raise the power compared with simple randomization. A study-independent biostatistician will be responsible for the randomization pattern generation. Participants will then be identified according to the inclusion/exclusion criteria. All participants within each township will receive the care allocated to their township hospital. For logistical reasons, the trial will start in one county (for both intervention and control clusters), then after two months be extended to the second county, and after four months to the third county. We expect participant recruitment to take two months in each county.

Participants will be identified from existing health records. The pilot study showed that each township hospital has an average of 500 eligible participants who may agree to participate in the trial. All eligible participants identified from existing health records will be asked to visit the township hospital, and will then be examined by the family doctor against the exclusion criteria. A well-trained nurse will consult participants on their willingness to participate and obtain informed consent.

### Ethical approval

The trial obtained ethical approval from the Research Ethics Committee of the University of Leeds, UK, on 8 October 2012 (reference HSLTLM/12/010) and the Ethics Committee of Zhejiang Provincial CDC, China, on 18 June 2012.

### Data collection

#### Primary outcome

CVD events will be collected from the Zhejiang provincial CVD surveillance system at 12 and 24 months after randomization. The system comprises data on all deaths and hospitalizations caused by heart disease and ischemic or hemorrhagic cerebrovascular disease reported by all levels of hospitals according to the WHO MONICA CVD events definition. The provincial CDC validates the accuracy of the reporting system through surveys on a regular basis.

In addition to the CVD surveillance system, participants in both arms will be asked retrospectively about stroke or heart attack diagnosed by hospitals at the county level or above at 12 and 24 months after randomization. Any hospital-confirmed reports would be amended in the surveillance system.

#### Secondary outcomes

Blood pressure, attendance rates, and rates of adherence to drugs will be collected using routine forms already in use in township hospitals, that is, the hypertensive and diabetic participant follow-up forms. On enrolment, the family doctor will measure the participant’s blood pressure using a standardized mercury sphygmomanometer after 5 minutes of seated rest, and measure body weight and height using standard measures. We have designed additional forms to record extra drug use in both intervention and control arms. Any serious adverse events will be recorded in a separate form during each follow-up consultation. In practice, participants need monthly prescriptions of drugs because the health insurance scheme only allows a single prescription to contain 1 month’s supply. Therefore, participants are likely to visit their family doctors every month. Adherence to booked appointments will be measured by examining attendance at booked appointments. Follow-up forms will be completed every quarter, collected from a computer-based system.

Participants’ blood profiles will be collected using sample surveys at 0 and 24 months after randomization. The surveys will comprise: 1) a questionnaire regarding participant demographics, socioeconomics, lifestyles, CVD history, current drug therapies if any, health service use, medical and related costs, and quality of life using the validated Chinese version of EQ-5D (EuroQol, Rotterdam, The Netherlands); and 2) a blood sample for measuring blood glucose and lipid profiles. Blood profiles will be measured with specifications of the fasting period, and will be collected according to the procedures outlined in the National Institutes of Health (NIH) guidelines [46] and Chinese guidelines [47]. Blood samples will be collected and transferred to the Zhejiang Provincial CDC. Standardized measurements, such as the enzymatic method, will be used.

#### Costing and cost-effectiveness analyses

An economic study from the societal perspective will be conducted to compare the cost-effectiveness of this intervention with usual care. The costs of the intervention over and above usual care (excluding research costs) will be collected, and used together with the above effectiveness/outcome measures to calculate the incremental cost-effectiveness ratio. Costs will include capital costs, such as training, other costs to strengthen the capacity of providers to deliver enhanced services, and recurrent costs, such as drugs and laboratory tests. Doctors’ salaries will not be included as no extra working time is sought in the intervention. Project record review and interviews with managers will be conducted to obtain cost data. Participant costs, including costs of drugs and other treatments, transportation, and loss of productivity, will be collected through a questionnaire survey, to be conducted as part of the sample survey at 0 and 24 months after randomization. Hospitalization and any loss of work days due to CVD events, as well as time costs associated with participants’ travel and waiting time will be recorded and converted into monetary forms. Markov models [48] will be used to simulate possible future states of the control and treatment arms, and to capture possible longer-term health outcomes (for example, additional years of life) and associated costs of care.

#### Process evaluation

A process evaluation will: describe the health system and service delivery context in which the intervention was delivered; examine recruitment processes, both at the cluster level (township hospital) and the individual level (patient); explore whether or not the intervention was delivered as intended, both at the cluster level (training) and the individual level (provider delivery); and explore the responses to the intervention both at the cluster level (managers and providers) and the individual level (patients and treatment supporters). Methods will include document review (for example, recruitment records, meeting minutes), observation of training and consultations, and interviews (for example, with CDC officials, hospital managers, family doctors, patients, and treatment supporters). Data will be collected at approximately 6 and 18 months into the trial. A sampling frame will be developed and participants will be purposively selected for inclusion from the above sampling sites.

### Analysis

This study is designed as a pragmatic trial of the care strategies within the primary healthcare context of China, rather than a therapeutic agent trial *per se*. Measures will be taken, however, to ensure a blinded outcome evaluation using: 1) the ‘PROBE’ design [49]; and 2) blinding those analyzing the outcome data to treatment status. The primary and some secondary outcomes are objective and not affected by participant or provider knowledge of treatment allocation. The primary outcomes are CVD events, such as strokes and myocardial infarction, which are objective outcomes. They are diagnosed by doctors at the county hospital who are not involved in the intervention and reported through the internet reporting system. Patients in both arms will be asked about CVD events, and non-reported events will be entered into the reporting system. In addition, the statistician will be blinded to which is the intervention arm. The secondary outcomes include drug use and healthy lifestyle endpoints, which may result in more variables in the intervention arm compared with the control arm. To avoid this as a leaking sign, we will generate a set of dummy data for additional health style endpoints to balance the data of two treatment groups; therefore, the independent statistician will not be able to distinguish which group is the intervention arm.

Analysis will be by intention-to-treat as the primary analysis and then per protocol, and will be conducted at the cluster level. The primary analysis will be undertaken on a dataset that includes all participants recruited in the study. The mean difference between the intervention and control outcome measures will be calculated. Potential prognostic factors will be controlled for to assess the effect of the intervention. In secondary analysis, mortality and morbidity of CVD events will be analyzed separately. In addition, the secondary analysis will also explore the relationship between adherence to drugs and primary or secondary outcomes. All outcomes will be analyzed at the end of the trial: no formal interim analysis is planned for this study. Safety data will be reviewed formally at 6 months of follow-up to ensure participants’ safety and integrity of the study.

A detailed data analysis plan is provided in Additional file 1.

## Discussion

Reducing CVD events requires long-term care that is proactive, patient-centred, community-based, and sustainable for patients with hypertension, diabetes, and related CVD conditions. Equitable delivery at a feasible cost is only possible through systems based on primary care [50,51]. The challenge, as outlined in the WHO package of essential non-communicable disease interventions [52], is how to implement a systematic, feasible, and effective model for primary healthcare in low resource settings.

This trial is not designed to compare therapeutic and health lifestyle interventions, but rather their combined effects within the context of China’s health policy and practice. Specific effects of either therapeutic and lifestyle interventions have been well documented in the literature. Rather, we will test the combined effects in realistic primary care practice as a pragmatic trial. Adherence rates will be a secondary outcome. We expect lower adherence rates for statins owing to their high cost. The sample size has been adjusted to detect a relatively small change of CVD risk due to possibly smaller numbers of patients agreeing to take the preventive drugs, and so would be the compliance rates of drugs (compared with the more usual hospital-based drug trials).

The is the first trial to date in developing countries to synthesize all current evidence on therapeutic and healthy lifestyle interventions to prevent CVD events in a primary care setting. Previous CVD prevention programs were mostly conducted through teaching hospitals in urban settings with very limited involvement of rural township hospitals (the primary care providers in rural China) [53]. A recent trial implemented by the George Institute in China differs from this one in terms of the population studied, drugs used, lifestyle intervention, and primary outcome (blood pressure rather than CVD rate reduction). The interventions fit within China’s national health reform programs, which are designed to strengthen primary care, essential public health tasks, and the rural health insurance scheme.

Through the trial, we hope to understand the barriers and facilitators that affect CVD control, and assess how to overcome implementation challenges and scale-up the package. Outputs will include a case management guide, training modules, and other materials that may be scaled-up in Zhejiang Province and other parts of China. These study materials also have the potential to be adapted to other low/middle income countries.

The success of the trial depends on the high uptake of the drugs and lifestyle advice prescribed. Challenges from both the participant and provider perspectives need to be addressed. The first challenge is related to the cost of medication. Drugs recommended in this trial are commonly available in primary healthcare practice in China. However, patients need to co-pay 70% of the cost of these drugs [54]. The cost challenge, especially due to the combined medication, may be offset by the increasing health awareness of chronically ill patients. A study in 2006 found that the majority of Chinese people believed that antihypertensive drugs were beneficial, and were willing to pay £50 per year to reduce a 5-year CVD risk of 35% or higher [55]. Furthermore, we estimated a smaller effect size for the pragmatic trial compared with that from hospital-based drug trials. The second challenge is people’s misperception of their health. The pilot study showed some participants were reluctant to take medicine or stopped medication when their symptoms diminished. This challenge can be addressed through regular health education and family treatment supporters in the intervention design. The third challenge is related to the primary healthcare providers’ capacity to implement the interventions. This will be addressed through innovative training sessions, such as participatory training, as well as training using the opportunity of routine refresher training sessions in the township hospitals. The quality of the trial will be ensured through enhanced supervision activities from the county’s CDC. The expected increase in prescription and regular use of preventive drugs in the intervention group will increase the risk of adverse effects, but in a population with a high CVD risk, the benefits outweigh the risks [11,36]. The family doctors will closely monitor the adverse effects based on the specific guidelines. Any serious adverse events will be documented and referred to county hospitals.

In both control and intervention sites, CVD events are diagnosed in hospitals at county level and above. All county or above hospitals use the same diagnostic guidelines required by the provincial CVD surveillance system. Given incomplete reporting of the electronic reporting system (currently missing around 10% to 15%), we will match reported CVD events against individuals enrolled in the trial using the health insurance number as the unified identifier. This will also be triangulated through participant-reported CVD events in both arms.

## Trial status

At the time of submission of the manuscript, the pilot study had just finished. The trial will start in September 2013 and the participants will be recruited during the first 2 months, September and October 2013. It is expected that the trial will last 24 months. The Trial Steering Committee and the Data Management and Ethical Committee of the trial were set up in June 2013. The sponsor of the trial is the China Program of the Communicable Disease Health Service Delivery (COMDIS-HSD) Research Consortium, which includes researchers from both the Chinese University of Hong Kong, China, and University of Leeds, UK.

## Abbreviations

CDC: Centre for Disease Control and Prevention; COMDIS-HSD: Communicable Disease Health Service Delivery; CV: Coefficient of variation; CVD: Cardiovascular disease; DFID: Department for International Development; Hb1Ac: Glycated hemoglobin; ICC: Intracluster correlation; MONICA: Monitoring of trends and determinants in cardiovascular disease; NIH: National Institutes of Health; RCT: Randomized controlled trial; TC: Total cholesterol; WHO: World Health Organization.

## Competing interests

The authors declare that they have no competing interests.

## Authors’ contributions

All authors made substantive contributions to the trial development and provided final approval for the manuscript. XW, GZ, ZZ, JY, and JW designed the trial and related studies, and drafted the manuscript. MY, WG, YY, CK, RK, and JT contributed to designing the trial and participated in the pilot study. MC and BZ contributed to the statistical issues in the RCT design and statistical analysis plan. SL contributed to the costing study. RK contributed to the process evaluation. SG critically reviewed the manuscript. XW and MY are co-principal investigators of the trial.

## Supplementary Material

Additional file 1 Statistical analysis plan. Click here for file
