# Supplementary material for: Cardiovascular disease risk reduction in rural China: a clustered randomized controlled trial in Zhejiang
Source: Trials. 2013 Oct 25;14:354. doi: 10.1186/1745-6215-14-354 (PMC4015636; doi:10.1186/1745-6215-14-354)
Supplement: Additional file 1 — Statistical analysis plan. [file 1745-6215-14-354-S1.pdf]

Statistic Analysis Plan

1. Trial Objectives

Cardiovascular disease (CVD) is the major killer in China. Despite government efforts, the majority of hypertensive and diabetic patients in China do not have proper treatment. Reducing CVD events requires long-term care that is proactive, patient centred, community based and sustainable. We designed a package of interventions to be implemented by family doctors of township hospitals in rural Zhejiang, China. This trial aims to explore whether such a systematic CVD risk reduction package can reduce CVD events of the population in the intervention arm compared with the usual care in the control arm and whether the package is cost effective, feasible to implement and scale up.

Primary objective

To explore whether such a systematic CVD risk reduction programme can reduce CVD events of the subjects in the intervention group compared with the usual care.

Design

It is a health service delivery trial that combines screening, treatment and life-style change interventions. It is designed as prospective, open-labelled, cluster randomized controlled trial with blinded data analysis. Based on information from the China hypertension control guideline and systematic reviews, a package of cardiovascular risk reduction interventions will be first piloted and then trialled in Zhejiang China. The package will include: 1) Health Education: smoking cessation, salt, sugar and oil reduction; 2) Proven Drugs: anti-hypertensive's, low dose aspirin, and a statin; 3) Adherence support for drug compliance, attendance and healthy lifestyle change; The intervention will be designed as job descriptions of family doctors in the areas and implemented within the primary care delivery system in Zhejiang China.

2. Sample Size

Sample size for the main study(primary outcome)
We expect our intervention to lead to a reduction of at least 20% in CVD event rates within two years. This is based on a conservative estimate from the results from meta-analyses [9, 11, 18, 37], a review of community based programmes in China [20], and recent trials [13, 14, 19, 40]. According to the baseline of our pilot study, the target population (50-74 years with ≥20% CVD risk and diabetes) has a CVD incidence rate of 5% within two years: we use this as an estimate of the CVD event rate in the usual care arm. In order to detect a 20% difference between the two arms with 90% power, using two-sided testing at the 5% level, and a moderate coefficient of variation (CV) of 0.15 [42]) with approximately 450 participants per cluster (using harmonic means to adjust for imbalances in cluster size [41]), we estimate 32 clusters per arm are needed [42, 43].

Sample size calculation for secondary outcomes of blood profiles

Due to resource limitations, we will collect participant blood samples to measure blood glucose, glycated haemoglobin and lipid profiles only on a subsample of participants. The survey will be conducted at 0th and 24th month after randomisation. The sample size is based on change in total cholesterol (TC) level, the blood profile indicator anticipated to show the smallest change of means (and hence expected to require the largest sample size). According to the pilot study, the mean and standard deviation of TC were 4.48mmol/L and 0.8526mmol/L respectively. We anticipate a reduction of 6% . In other similar studies, a TC reduction of 16-20% was observed [14, 44]. In our pilot study, a third of participants took statin. We therefore assume that we will get 18%/3 reduction in TC, ie 6% in the intervention arm. We estimate we need to increase the sample size by a factor of 2 to allow for clustering, with an intra-cluster correlation (ICC) of 0.01 and a maximal 100 participants per cluster. Clusters will be chosen randomly from each arm and sampling will be done within the required clusters. With 90% power and using two-sided testing at the 5% level, 6 clusters are required in each arm, allowing for a loss to follow up rate of 10% [41, 55].

3. Randomisation

All township hospitals with available health records in the three countries in Shaoxing, Zhejiang will be selected except the pilot site. Written informed consent will be sought from each township hospital. Written informed consent will also be collected from each of the participants in township hospitals before screening.

Townships will be randomised (without stratification) to intervention or control in a 1:1 ratio. No blinding will be done in this study. The sensitivity analysis shows that stratified randomisation does not significantly raise the power compared with simple randomisation. A study-independent biostatistician will be responsible for the randomization pattern generation. Participants will then be identified according to the inclusion/exclusion criteria. All participants within each township will receive the care allocated to their township hospital. For logistical reasons, the trial will start in one county (for both intervention and control clusters), then after two months be extended to the second county, and after four months to the third county. We expect participant recruitment to take two months in each county.

Participants will be identified from existing health records. Our pilot study showed that each township hospital has an average of 500 eligible participants who may agree to participate in the trial. All eligible participants identified from existing health records will be asked to visit the township hospital, and will then be examined by the family doctor against exclusion criteria. A well-trained nurse will consult participants on their willingness to participate and obtain informed consent.

4. Definition of terms

CVD events include coronary event and stroke event.
The coronary events has the follow categories: 1) definite acute myocardial infarction; 2) possible acute myocardial infarction or coronary death; 3) ischaemic cardiac arrest with successful resuscitation not fulfilling criteria for definite or possible myocardial infarction; 4) no acute myocardial infarction or coronary death; 5) fatal cases with insufficient data, subsequently called "unclassifiable deaths" in collaborative MONICA publications.

Stroke is defined as rapidly developed clinical signs of focal (or global1) disturbance of cerebral function lasting more than 24 hours (unless interrupted by surgery or death), with no apparent cause other than a vascular origin: it includes patients presenting clinical signs and symptoms suggestive of subarachnoid haemorrhage, intracerebral haemorrhage or cerebral ischaemic necrosis. It does not include transient cerebral ischaemia or stroke events in cases of blood disease (e.g. leukaemia, polycythaemia vera), brain tumour or brain metastases. Secondary stroke caused by trauma should also be excluded [10].

5. Outcomes

Primary outcome(s)

The primary outcome is the incidence of severe CVD events over 24 months of follow-up. CVD events are defined according to the WHO MONICA definitions, including: 1) acute coronary events; 2) acute myocardial infarction, sudden cardiac death and other deaths due to coronary and vascular disease; 3) ischemic or hemorrhagic cerebrovascular events. Minor CVD events such as chronic cerebral arteriosclerosis and transient ischemic attacks are not included because these events do not usually end in hospitalisation and are often not reported. Participants who cannot be contacted after three attempts (either by phone call, emails, or letters) by the 24th month after randomisation will be deemed as lost to follow up. Person years will be used to calculate CVD event rates for participants whose CVD events outcomes are recorded. If sufficient information about CVD events is available, the calculation of CVD events outcomes will also cover the last contact time for the loss-to-follow-up cases and mortality cases that were not due to CVD events.

Secondary outcome(s)

(1) Mean systolic and diastolic blood pressures of participants; (2) time to the first reported CVD event, mortality, and morbidity of CVD events during the trial period of 24 months; (3) mean change in blood glucose and glycated haemoglobin (Hb1Ac); (4) mean change in serum total cholesterol and low density lipoprotein; (5) adherence to booked appointments, using the denominator of all participants registered, including defaulters; (6) self-reported adherence to drugs and healthy lifestyle change (e.g. smoking cessation rates – for participants who smoke at randomisation, the percentage who smoke less than one cigarette a week at the 24th month after randomisation); (7) cost-effectiveness; and (8) feasibility measures.

Population for follow-up

All study participants will be treated and managed under township hospitals by family doctors for 24 months.

Other important information

Information of the subjects will be collected at the point of recruitment include characteristics of participants (name, address, ID, gender, age), whether recruit or not, reasons for not recruiting. Patient name, ID and address will be collected for the routine follow up purpose, but not to be used in the general data coding plan to protect patient identities. Information of follow up in both arms, including prescription of drugs, adherence to drugs and lifestyle changes, number of participants followed up and the reasons for not following up, will be collected in each quarter.

6. Data collection

Primary outcome

CVD events will be collected from the Zhejiang provincial CVD surveillance system at the 12th and 24th month after randomisation. The system comprises data on all deaths and hospitalisations caused by heart disease and ischemic or haemorrhagic cerebrovascular disease reported by all levels of hospital according to the WHO MONICA CVD events definition. The provincial CDC validates the accuracy of the reporting system through surveys on a regular basis.

In addition to the CVD surveillance system, participants will be asked about stroke or heart attack diagnosed by hospitals at the county level or above at 12th and 24th months after randomisation. Any hospital confirmed reports will be amended in the surveillance system.

Secondary outcomes

Blood pressure, attendance rates and rates of adherence to drugs will be collected using routine forms already in use in township hospitals, i.e. the hypertensive and diabetic participant follow-up forms. On enrolment, the family doctor will measure the participant’s blood pressure using a standardized mercury sphygmomanometer after 5 minutes of seated rest, and body weight and height using standard measures. We have designed additional forms to record extra drug use in both intervention and control arms. Any serious adverse events will be recorded in a separate form during each follow-up consultation. In practice, participants need monthly prescriptions of drugs because the health insurance scheme only allows a single prescription to contain one month’s supply. Therefore, participants are likely to visit their family doctors every month. Adherence to booked appointments will be measured by examining attendance at booked appointments. Follow-up forms will be completed every quarter, collected from a computer-based system.

Participants' blood profiles will be collected using sample surveys at the 0th and 24th months after randomisation. The surveys will comprise: (1) a questionnaire regarding participant demographics, social economics, lifestyles, CVD history, current drug therapies if any, health service use, medical and related costs, and quality of life using the validated Chinese version of EQ-5D; and (2) a blood sample for measuring blood glucose and lipid profiles. Blood profiles will be measured with specifications of the fasting period and will be collected according to the procedures outlined in the US national guidelines [45] and Chinese guidelines [46]. Blood samples will be collected and transferred to the Zhejiang Provincial CDC. Standardised measurements such as the enzymatic method will be used.

Costing and cost-effectiveness analyses

An economic study from the societal perspective will be conducted to compare the cost-effectiveness of this intervention with usual care. The costs of the intervention over and above usual care (excluding research costs) will be collected, and used together with the above effectiveness/outcome measures to calculate the incremental cost-effectiveness ratio. Costs will include capital costs such as training, and other costs to strengthen the capacity of providers to deliver enhanced services, and recurrent costs such as drugs, and laboratory tests. Doctors' salaries will not be included as no extra working time is sought in the intervention. Project record review and interviews with managers will be conducted to obtain cost data. Participant costs, including costs of drugs and other treatments, transportation, loss of productivity, etc, will be collected through a questionnaire survey, to be conducted as part of the sample survey mentioned above at 0th and 24th months after randomisation. Hospitalization and any loss of work days due to CVD events, as well as time costs associated with participants' travel and waiting time will be recorded and converted into monetary forms. Markov models [47] will be used to simulate possible future states of the control and treatment arms, and to capture possible longer-term health outcomes (e.g. additional years of life) and associated costs of care.

Process evaluation

A process evaluation will describe the health system and service delivery context in which the intervention was delivered; examine recruitment processes, both at the cluster level (township hospital) and the individual level (patient); explore whether or not the intervention was delivered as intended, both at the cluster level (training) and the individual level (provider delivery); and explore the responses to the intervention both at the cluster level (managers and providers) and the individual level (patients and treatment supporters). Methods will include document review (e.g. recruitment records, meeting minutes), observation of trainings and consultations, and interviews (e.g. with CDC officials, hospital managers, family doctors, patients and treatments supporters). Data will be collected at approximately 6 months and 18 months into the trial. A sampling frame will be developed and participants will be purposively selected for inclusion from the above sampling sites.

7. Analysis

Analysis Populations

Intention-to-treat (ITT) population is defined as all successfully recruited subjects.
Survey (SY) population is defined as all randomly selected enrolled subjects who consent to the survey (offering data).
Evaluable (EV) population is defined as subjects who complete the 24th month study period with records of CVD events in either the CVD surveillance system or the follow-up. The EV set will mainly serve for the primary analysis purpose. The ITT set and SY set will be adopted for other analysis.

Final Analysis and Reporting

No formal interim analysis is planned for this study. Any changes to the statistical analysis plan will be documented in the final analysis report. The significance tests will be two-sided and the cut-off point for statistical significance was set at the 5% level.

Analysis Software

All analysis will be performed using SAS® and SPSS® software.

Missing Data

No imputation is considered in the analysis.

Baseline Data

Baseline assessment data such as demographics (sex, age, marital status, occupation, education level, and family income) and blood pressures for patients will be analyzed descriptively by two study groups. Additional baseline survey data: socio-economic, healthy lifestyles, CVD history, current drug therapies, health service use, medical expenditure, and quality of life, and blood sample data will also be summarized descriptively by treatment arms and townships. Continuous measures such as blood pressure will be reported as means and standard deviations, while the categorical data such as lifestyle changes will be reported as numbers and proportions by times.

Primary Analysis

The primary endpoint, the difference of CVD event rate between the intervention arm and the control arm during the trial period of 24 months, will be carried out at both cluster (township) and individual levels. Descriptive statistics will be used to summarize for the two study arms. Statistical inference will be drawn on township-level summaries and individual level data [16]. The CVD event rate ratio with corresponding 95% confidence intervals based on township-level summaries will be provided for the two study arms. To account for individual level covariates, the CVD event rate will be compared by multilevel log linear models (SAS PROC MIXED) [19]. Adjusted t-test will also conducted to compare the CVD event rate

between two arms accounting for the design effect [19]. Poisson mixed effect model will be adopted for adjusting random cluster effect and additional covariates.Potential confounders such as baseline blood pressures and significant demographic variables would be included in models.

Secondary Analysis

Secondary analysis will also be carried out for both cluster (township) and individual levels. As for the cluster-level summaries, the cluster mean changes of the main secondary outcomes: systolic and diastolic blood pressure from baseline to the 24th month after the randomization between the intervention group and the control group will be compared by an unpaired t-test [16]. Multilevel linear models (SAS PROC MIXED) will be adopted in order to adjust the individual effects. Individual level analysis will be conducted by a mixed effect model with adjusting random cluster effect and additional covariates. Potential significant confounders will be included in models. Descriptive statistics such as quarter mean percentage changes will be showed at an individual level. 95% confidence intervals of means will be provided for treatments and measurement time points.

In the study, we will compare the difference of the time to the first reported CVD event, mortality, and morbidity of CVD events during the trial period of 24 months. The unadjusted time to events will be analyzed with the use of Kaplan-Meier curves and log-rank tests. Cox proportional hazard mixed effect models (SAS PROC PHREG and PROC NLMIXED) will be used to study the hazard ratio between the intervention arm and the control arm adjusted with the cluster random effect and the effect from the baseline significant confounders. Corresponding 95% confidence interval will be provided.

For other secondary endpoints: blood glucose, total cholesterol, and low density lipoprotein, the mean percentage changes with 95% confidence intervals (adjusted with design effect [19]) will be calculated using the SY set subjects by treatment arms and township population sizes from the 0th month to the 24th month after randomisation. Linear models with repeated measures will be adopted to compare the treatment effect adjusted with time effects. Other confounders will be included in the models.

For those subjects received study intervention, mean adherence rate to booked appointments and the self-reported adherence rate to drugs and healthy lifestyle change will be calculated. Effect of adherence on the risk of CVD and other secondary outcomes will also be investigated in the models.

Mortality and morbidity of CVD events will be analysed separately; using a method similar to the primary analysis.

In the cost effectiveness analysis, intervention effects and average costs collected from patient questionnaire surveys will be calculated and they will be part of the

Markov model inputs. Probability distributions of input parameters will be tested in a sensitivity analysis to the model in order to explore the possible range (or 95% non-parametric confidence interval) of health outcomes as well as the incremental cost effectiveness ratio.

Subgroup Analysis

Subgroup analysis to study endpoints will be considered according to different strata such as: adherence rates (high and low), population size (high, middle, and low), and drug therapies (anti-hypertensive drugs and anti-diabetic drugs).

Safety Analysis

Safety analysis will base on ITT principle. All serious adverse events (SAE) data collected during the study period will be listed.

Other Study Outcomes

Ongoing treatments will be analysed descriptively and listed for a comparison among study groups. Descriptive statistics will be used to demonstrate the difference of quality of life (QOL) between study groups. Difference of QOL data between two treatment groups from the 0th month and the 24th month will be tested by a paired t-test.

8. References

1. Law MR, Wald NJ, Morris JK, Jordan RE: **Value of low dose combination treatment with blood pressure lowering drugs: analysis of 354 randomised trials**. *BMJ* 2003, **326**(7404):1427.
2. Wald NJ, Law MR: **A strategy to reduce cardiovascular disease by more than 80%**. *BMJ* 2003, **326**(7404):1419.
3. Murray CJ, Lauer JA, Hutubessy RC, Niessen L, Tomijima N, Rodgers A, Lawes CM, Evans DB: **Effectiveness and costs of interventions to lower systolic blood pressure and cholesterol: a global and regional analysis on reduction of cardiovascular-disease risk**. *Lancet* 2003, **361**(9359):717-725.
4. Wang X, Qin X, Demirtas H, Li J, Mao G, Huo Y, Sun N, Liu L, Xu X: **Efficacy of folic acid supplementation in stroke prevention: a meta-analysis**. *Lancet* 2007, **369**(9576):1876-1882.
5. Yusuf S, Pais P, Afzal R, Xavier D, Teo K, Eikelboom J, Sigamani A, Mohan V, Gupta R, Thomas N: **Effects of a polypill (Polycap) on risk factors in middle-aged individuals without cardiovascular disease (TIPS): a phase II, double-blind, randomised trial**. *Lancet* 2009, **373**(9672):1341-1351.
6. Malekzadeh F, Marshall T, Pourshams A, Gharravi M, Aslani A, Nateghi A, Rastegarpanah M, Khoshnia M, Semnani S, Salahi R *et al*: **A pilot double-blind randomised placebo-controlled trial of the effects of fixed-dose combination therapy ('polypill') on cardiovascular risk factors**. *Int J Clin Pract* 2010, **64**(9):1220-1227.

7. Mao G, Hong X, Xing H, Liu P, Liu H, Yu Y, Zhang S, Jiang S, Wang X, Xu X: **Efficacy of folic acid and enalapril combined therapy on reduction of blood pressure and plasma glucose: a multicentre, randomized, double-blind, parallel-controlled, clinical trial**. *Nutrition* 2008, **24**(11-12):1088-1096.
8. Hayes RJ, Bennett S: **Simple sample size calculation for cluster-randomized trials**. *International Journal of Epidemiology* 1999, **28**(2):319-326.
9. Kaczorowski J, Chambers LW, Karwalajtys T, Dolovich L, Farrell B, McDonough B, Sebaldt R, Levitt C, Hogg W, Thabane L *et al*: **Cardiovascular Health Awareness Programme (CHAP): A community cluster-randomised trial among elderly Canadians**. *Preventive Medicine* 2008, **46**(6):537-544.
10. WHO MONICA Project. MONICA Manual. http://www.thl.fi/publications/monica/manual/index.htm
11. NIH: **National Cholesterol Education Programme. 2009**. Bethesda, Maryland: National Heart, Lung and Blood Institute, National Institute of Health; 2009.
12. Liu L, Wang W, Yao C, China MoH: **China hypertension prevention and control guideline: for primary care**. *Chinese Journal of Hypertension* 2009, **18**(1):11-30.
13. Munro B: *Statistical methods for health care research*. 4th edition. Philadelphia: Lippincott; 2001.
14. Sonnenberg F, Beck J: **Markov models in medical decision making: a practical guide**. *Med Decis Making* 1993, **13**:322-338.
15. Hayes RJ and Bennett S: **Sample Size Formulae for Cluster-randomized Designs**. *Int j epidemi* 1999, **28**:319-326.
16. Hayes RJ, Moulton LH: **Cluster Randomised Trials**. *Chapman & Hall/CRC*; 2009.
17. Study of cholesterol lowering with simvastatin in 20,536 high-risk individuals: a randomised placebo-controlled trial. Lancet, 2002; 360: 7–22.
18. F. Malekzadeh, T. Marshall, A. Pourshams, et al. **A pilot double-blind randomised placebo controlled trial of the effects of fixed-dose combination therapy ('polypill') on cardiovascular risk factors.** *The international Journal of clinical practice*, 2010, 64(9): 1220-1227.
19. CAMPBELL, Marion K., et al. **Analysis of cluster randomized trials in primary care: a practical approach**. *Family practice*, 2000, 17.2: 192-196.
20. Liu M, Wu B, Wang WZ, Lee LM, Zhang SH, Kong LZ: **Stroke in China: epidemiology, prevention, and management strategies**. *Lancet neurology* 2007, **6**(5):456-464.
21. **MRC/BHF Heart Protection Study of cholesterol lowering with simvastatin in 20,536 high-risk individuals: a randomised placebo-controlled trial**. *Lancet* 2002, **360**(9326):7-22.
22. Kerry SM, Bland JM: **The intracluster correlation coefficient in cluster randomisation**. *BMJ* 1998, **316**:1455.

9. Appendices

Templates of Tables and Figures

Templates of Tables and Figures will be provided upon the confirmation case report forms and questionnaire.
